# Metabolic Analysis of Adaptation to Short-Term Changes in Culture Conditions of the Marine Diatom *Thalassiosira pseudonana*

**DOI:** 10.1371/journal.pone.0067340

**Published:** 2013-06-14

**Authors:** Mariusz A. Bromke, Patrick Giavalisco, Lothar Willmitzer, Holger Hesse

**Affiliations:** Department of Molecular Physiology, Max Planck Institute of Molecular Plant Physiology, Potsdam, Germany; Instituto de Biología Molecular y Celular de Plantas, Spain

## Abstract

This report describes the metabolic and lipidomic profiling of 97 low-molecular weight compounds from the primary metabolism and 124 lipid compounds of the diatom *Thalassiosira pseudonana*. The metabolic profiles were created for diatoms perturbed for 24 hours with four different treatments: (I) removal of nitrogen, (II) lower iron concentration, (III) addition of sea salt, (IV) addition of carbonate to their growth media. Our results show that as early as 24 hours after nitrogen depletion significant qualitative and quantitative change in lipid composition as well as in the primary metabolism of *Thalassiosira pseudonana* occurs. So we can observe the accumulation of several storage lipids, namely triacylglycerides, and TCA cycle intermediates, of which citric acid increases more than 10-fold. These changes are positively correlated with expression of TCA enzymes genes. Next to the TCA cycle intermediates and storage lipid changes, we have observed decrease in N-containing lipids and primary metabolites such as amino acids. As a measure of counteracting nitrogen starvation, we have observed elevated expression levels of nitrogen uptake and amino acid biosynthetic genes. This indicates that diatoms can fast and efficiently adapt to changing environment by altering the metabolic fluxes and metabolite abundances. Especially, the accumulation of proline and the decrease of dimethylsulfoniopropionate suggest that the proline is the main osmoprotectant for the diatom in nitrogen rich conditions.

## Introduction

Diatoms are found in almost all aquatic habitats and are responsible for 20% of the global primary production [1,2]. Through their effective photosynthetic fixation of CO_2_ and the formation of organic compounds diatoms play a major role maintaining the food chain in the sea. Furthermore, diatoms contribute to biogeochemical cycling of carbon through their sedimentation after death, thus precluding CO_2_ from the atmosphere. Current estimates of uptake and conversion of CO_2_ suggest that changes of the global climate have also severe implications on diatoms [3]. Thus, the increase of atmospheric CO_2_ content and the resulting global warming place diatoms into the focus of several research projects [4].

Diatoms are heterokont algae. Their nuclear and plastidial genetic material was shaped by two events of endosymbiosis in the history of diatoms evolution [5]. Analysis of the genome sequence of *Thalassiosira pseudonana* revealed the presence of a whole suite of genes coding enzymes of the urea cycle, suggesting the importance of urea metabolism for nutrition of diatoms [6]. Biological and physical processes in the ocean greatly affect spatial and temporal nitrogen availability in marine environments. Diatoms are able to utilize a variety of inorganic (NO_3_^-^, NH_4_^+^) and organic (urea, amino acids) nitrogen sources adjusting their N metabolism to the available nutrients [7,8]. The nitrogen-limitation response in *Thalassiosira pseudonana* was analyzed on the level of transcription by Mock et al. [9] while Hockin et al. analysed the proteomic as well as the changes in free amino acids [10].

Iron is a growth-limiting element for all organisms and particularly for growth of photosynthetic algae, as it is a necessary component of the photosynthetic apparatus and mitochondrial electron transport chain. To cover the cellular demand for iron diatoms like *Thalassiosira pseudonana* seem to utilize a ferroxidase/permease uptake system, in which Fe^3+^ ions are reduced at the cell surface, followed by the coupled oxidation of Fe^2+^ to Fe^3+^ before importing them by a permease [11]. Iron limitation in diatoms leads to reduced synthesis of chlorophyll and a significant reduction of the efficiency of photosynthesis [12] as well as slower rate of nitrogen assimilation [13].

Diatoms have received special attention as a potential resource for the production of bioenergy. The major membrane lipids are the glycosylglycerides (e.g. monogalactosyldiacylglycerol, digalactosyldiacylglycerol and sulfoquinovosyldiacylglycerol), which are enriched in the chloroplast, together with significant amounts of phosphoglycerides, which mainly reside in the plasma membrane and many endoplasmic membranes [14]. However, under stress and nutrient starvation conditions biosynthesis of neutral lipids is enhanced. Thus, some algae can accumulate up to 50% of their dry weight triglycerides [14]. With respect to biodiesel, in addition to the quantity, the structural composition of TAG’s is important, as different fatty acids have different properties as components of fuel [15,16].

Diatoms are capable to swiftly adapt to changing nutrients conditions. This is observed especially in the upwelling environments, where nutrient-rich water is brought to the surface, diatoms show remarkable efficiency in the uptake of growth limiting nutrients such as silica, iron and nitrogen [17–20].

In the presented study we performed an extensive study of the swift adaptation of algae to changing environments. To this end we have focused on the response of the planktonic diatom *Thalassiosira pseudonana* - a ubiquitous, centric diatom species to short-termed limitation of nitrogen and lower iron concentration. In addition, we have exposed the algae to increased concentrations of carbonate and sea salts. The additional carbonate was given in order to explore the response to higher concentrations of biocarbonate and in consequence CO_2_. Addition of sea salt represents a rather artificial experiment which was motivated by the hope to learn more about compatible solute formation in *T. pseudonana*. The responses to these environmental changes were followed on the metabolic (primary metabolites) and lipidomic level, and were supported by profiling of transcripts of selected genes. Next to the broad scale metabolic profiling, we have focussed on the measurements of a single, highly relevant compound, namely dimethylsulfoniopropionate, which represents an important sulfur-containing metabolite produced by many algae.

## Results and Discussion

In the course of the performed experiments, diatom cells were transferred to four test media (low nitrogen, low iron, high carbonate, and high sea salt). Twenty-four hours after the medium shift, the cells were harvested and levels of extracted analytes as well as the abundance of selected transcripts was analysed. The data were normalized and the obtained values were compared to the values of the diatoms transferred to the control, f/2 medium. In recent years several reports on diatoms metabolomics were published, with descriptions of metabolic profiles of benthic diatoms *Phaeodactylum* *tricornutum* [21], *Cocconeisscutellum* [22] or planktonic *Skeletonema* *marinoi* [23]. However, to our knowledge this publication is the first metabolic analysis of such breadth on *Thalassiosira pseudonana.*

**Figure 1:**
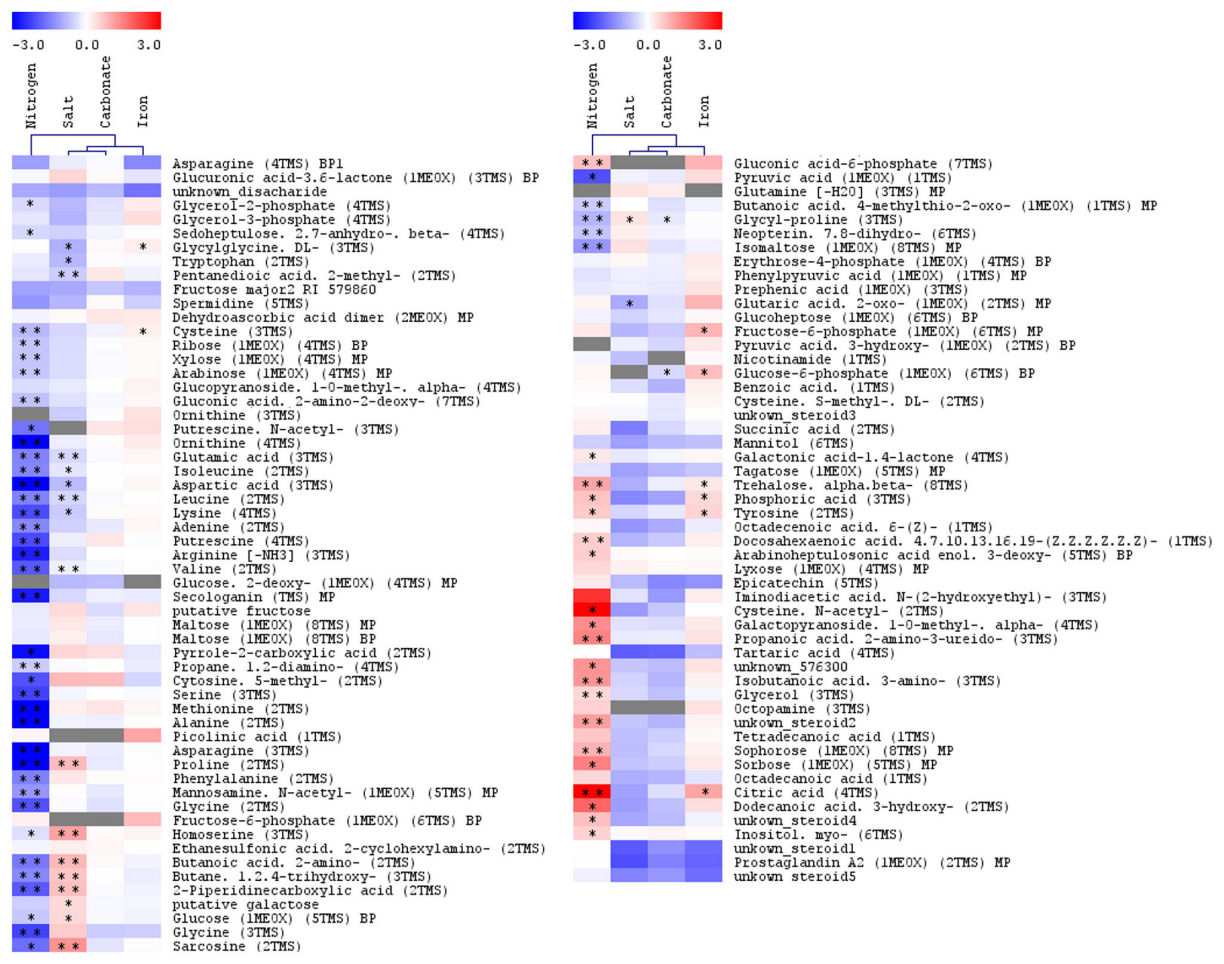
Heat-map of metabolic changes in*Thalassiosira pseudonana* treated in four conditions for 24 hours. Pearson correlation was used to cluster the results. Intensity of colours represents log_2_-transformed ratios of measured mean (n=5) analyte’s intensity to its respective mean value in the control conditions. Analytes, which could not be measured in more than 3 samples, were marked grey. Asterisks mark t-test P-value, where “**” marks P < 0.01 and “*” marks P < 0.05. Note, this is one heat-map, which has been presented in two blocks of data.

**Figure 2:**
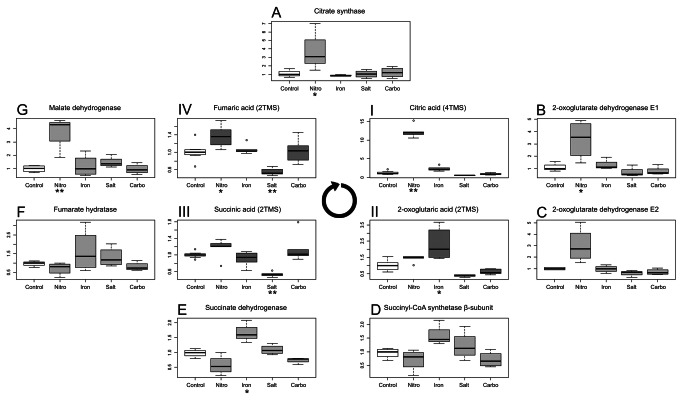
Changes in the TCA cycle intermediates and expression of related genes. Three inner boxplots represent levels of (I) citric acid, (II) 2-oxoglutaric acid (III) succinic acid and (IV) fumaric acids measured in cells of *T. pseudonana* grown in different conditions: nitrogen limitation (Nitro), lowered iron concentration (Iron), salt addition (Salt), carbonate addition (Carbo). Values on the plots represent median values (horizontal line) of normalized peak intensity divided by median for the control samples. Outer boxplots represent relative expression of TCA-enzymes genes. Values on the plots represent median values (horizontal line) of expression divided by median for the control samples. Asterisks below a treatments name mark a statistical significant change with p-value <0.05 * or p-value < 0.01 ** in Tukey’s Test. The round arrow indicates the direction of the TCA cycle.

Figure 1 summarizes the metabolic responses of the 97 compounds measured by GC-TOF MS. As can be seen from the Figure 1, especially nitrogen deficiency and sea salt stress produced major and distinct metabolic phenotypes. Even though the nitrogen deprived *Thalassiosira pseudonana* cells display only slightly reduced growth rate after 24 hours, a strongly reduced growth was observed after 48 hours (data not shown). The same holds true for diatoms grown in lower iron concentration medium, as well as the sea salts treated cultures. As we are interested in the early response of *T. pseudonana* to changing environments, we have taken samples already after 24 hours in the new medium. The metabolomics analysis shows a significant reduction in the content of many nitrogen-containing metabolites such as polyamines and amino acids. We have observed that the levels of methionine, proline and aspartic acid, which all are amino acids made from intermediates of the TCA cycle (oxaloacetate and 2-oxoglutaric acid), were most strongly reduced by 15-, 16- and 8.5-fold, respectively. Other amino acids including the branched-chain amino acids (leucine, isoleucine, valine), the sulphur-containing cysteine and the aromatic tyrosine were also significantly reduced. Also the level of non-proteinogenic amino acid ornithine (derived directly from glutamate) displayed a more than 8-fold reduction (Figure 1). These observations suggest a general reduction in the synthesis of nitrogenous compounds, which is in line with the results previously described by Hockin et al [24], who reported that exposure of *Thalassiosira pseudonana* to reduced nitrogen leads to a reduction in cellular protein and amino acid content. Further significant changes have been observed for several intermediates of the TCA cycle, including citric acid, 2-oxoglutaric acid, fumarate and succinic acid (Figure 2; I, II, III, IV). Interestingly, comparable results were also reported for *Chlamydomonas reinhardtii*, which after 24 hours of nitrogen deficiency, have shown reduced levels for most nitrogen containing compounds [25].

The addition of carbonate, which leads to increase of available C in the culture medium, had the mildest, almost neglectable, effect on the metabolite composition of *Thalassiosira pseudonana* (Figure 1). For only one compound (glucose-6-phosphate) we have observed a significant reduction.

Increasing the salt concentration instead, led, similarly to the depletion of nitrogen, to changes in several amino acid levels. A significant reduction was observed for the branched-chain amino acids valine, leucine, isoleucine, the aromatic amino acid tryptophan, oxaloacetate-derived aspartate and lysine, and 2-oxoglutaric acid-derived glutamate. Proline, which is a direct product of glutamate, showed the opposite behaviour and increased 1.65-fold over the control (Figure 1). This observation suggests that, similar to other organisms, *Thalassiosira pseudonana* could use proline as an osmoprotectant.

Another interesting metabolic pattern concerns sarcosine, which showed a major increase of more than 2-fold under the salt stress (Figure 1). Sarcosine is a degradation intermediate of glycine betaine, which in turn is regarded as an osmolyte for cells of *Thalassiosira pseudonana* [26] and probably its levels represents a surrogate for glycine betaine cellular content. Thus, the increased levels of sarcosine and proline indicate the stronger need for osmotic adjustment under the increased salt concentrations. In contrast to its increase under the elevated salt concentration conditions, sarcosine content is greatly reduced under nitrogen limiting growth conditions (Figure 1). This is similar to many other N-containing metabolites.

Hence, iron is a major limitation for growth of diatoms in the oceans, We have lowered the iron concentration for 24 hours in cultures of *Thalassiosira pseudonana* to analyse the more immediate effect of this condition on metabolism. Significant increase was observed for some amino acids, namely tyrosine and cysteine (Figure 1), which are derived from phosphoenolpyruvate and 3-phosphoglycerate, respectively. Additionally, sugar phosphates (glucose-6-phosphate, fructose-6-phosphate) and free phosphate were also increased significantly (Figure 1), as well as the TCA cycle intermediates citric acid and 2-oxoglutaric acid. Interestingly, this response pattern resembles, to some extent, the response described by Boelling et al. [25] for iron-depleted *Chlamydomonas reinhardtii*. The availability of iron, although reduced by omitting this element in prepared medium, was probably big enough to sustain *T. pseudonana* growth for 24 hours. The sources of iron ions could be a contamination of salts and equipment used for preparation of media as well as residual iron from the starting medium, which was transferred with the diatoms to the low-iron test medium. This can explain the mild effect of the low iron conditions on the growth and metabolic profiles observed in this study.

**Figure 3:**
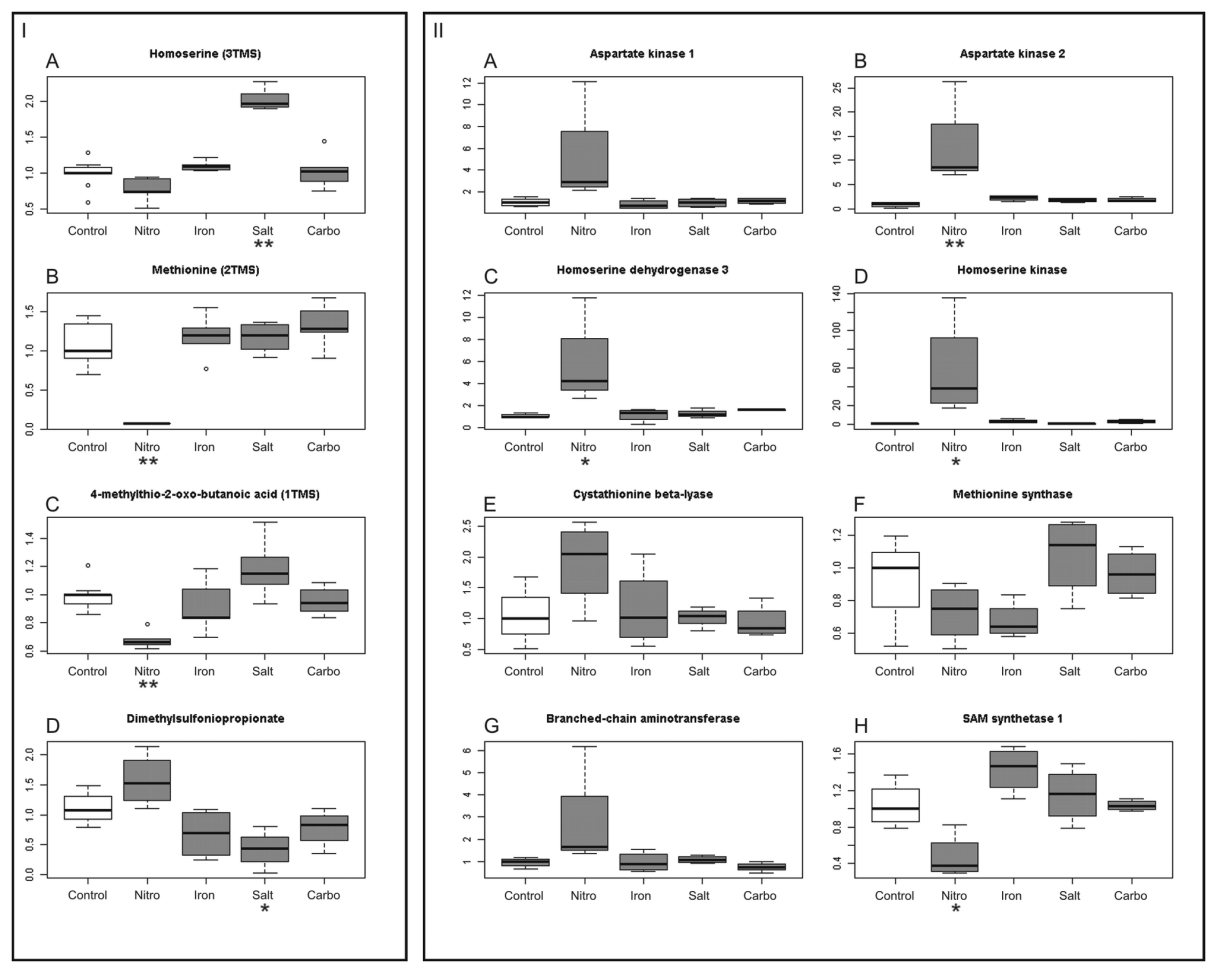
Changes of selected metabolite levels and gene expressions in*Thalassiosira pseudonana*. Panel I represents changes of homoserine, methionine, 4-methylthio-2-oxobutyrate and DMSP levels in *Thalassiosira pseudonana* cultivated in four different conditions: nitrogen limitation (Nitro), lowered iron concentration (Iron), salt addition (Salt), carbonate addition (Carbo). Values on the boxplots represent median values (horizontal line, n=5, n=4 in case of DMSP) of normalized peak intensity divided by median for the control samples. Panel II represents changes in gene expression of genes from biosynthesis pathway of aspartate-family amino acids. Values on the plots represent median values (horizontal line, n=4) of normalized transcript abundance divided by median for the control samples. Asterisks below a treatments name mark a statistical significant change with p-value <0.05 * or p-value < 0.01 ** in Tukey’s test.

Dimethylsulfoniopropionate (DMSP) is an important metabolite produced by diatoms, which is discussed in terms of biogeochemical cycling of sulfur. Once DMSP is released from an algal cell, it becomes a precursor for the volatile dimethylsulfide, which is the link between the oceanic and atmospheric sulfur [27]. It is assumed that cellular dimethylsulfoniopropionate functions in the osmo-regulation in form of a compatible solute in similar way as glycine betaine or proline [28]. Moreover, DMSP functions as a cryoprotectant [29] and an antioxidant [30]. Due to this importance for algal cells, we have decided to monitor the levels of DMSP under all four test conditions. As shown in Figure 3 I, DMSP significantly decreases under salt stress, whereas under nitrogen deprivation it increases, as described previously by Keller et al. [26]. DMSP in algae is synthesized from methionine in four steps [31]. In the first step of its synthesis, the deamination of methionine is probably catalysed by a transaminase [31]. To validate this assumption we decided to monitor the expression of the branched chain amino acid transferase (Prot ID260934), which shows high similarity to BCAT4 – an *Arabidopsis thaliana* aminotransferase, which catalyses the first synthesis step of Met-derived glucosinolates [32]. Comparisons of medians between control and N-starved diatoms suggest an increased expression of this gene, which is in agreement with findings by Mock et al. [9]. The level of the product of the transaminase reaction and an intermediate in DMSP synthesis, 4-methylthio-2-oxobutyrate (MTOB) was reduced more than 7-fold in nitrogen-starved *Thalassiosira pseudonana* (Figure 3 I).

Since this transcriptional response matched the metabolic observations we decided to validate whether the changes observed in the metabolite levels are also reflected on the level of gene expression. For this purpose we monitored the expression of several amino acid biosynthesis and TCA cycle enzymes genes via qRT-PCR. The most significant changes have nbeen observed in diatoms from the nitrogen-depleted conditions. In these conditions higher levels of RNA encoding citrate synthase, two subunits of α-oxoglutarate dehydrogenase and malate dehydrogenase have been observed (Figure 2: A, B, C, G). This confirms the metabolite measurements and extends previous reports [9,10]. In contrast to the behaviour of most amino acids, which decreased under nitrogen deprivation, an increased expression of most genes of the aspartate family pathway was observed. We could see, that genes encoding aspartate kinases, aspartate semialdehyde dehydrogenase, homoserine dehydrogenase and homoserine kinase, all have shown a significant increase (Figure 3 II A, B, C, D). Interestingly, transcript levels for the methionine synthase gene, even though content of this metabolite was significantly reduced (Figure 3 I), showed no significant change (Figure 3 II F). Low availability of methionine in nitrogen-starved diatoms seems to be connected with expression of genes which products utilize this amino acid. The expression of S-adenosylmethionine (SAM) synthetase 1 (Figure 3 II H) was halved in this conditions. Moreover, a tentative reduction of SAM synthetase 2 expression was observed as well (Table S1). The expression levels of discussed genes are also presented in the Table S1.

Taken together, the changes in metabolite and transcript levels observed in diatoms under the nitrogen-limiting conditions can be interpreted as a counter-strategy of the cells to supply the needed amino acids. This strategy is based on an increase of both, the gene expression and the metabolite levels of the TCA cycle (supposed to supply the carbon skeleton for amino acid biosynthesis) and the amino acid biosynthesis pathways, to be able to maintain the delicate levels balance in amino acids concentrations.

### Lipidomics

As described in the introduction, diatoms are discussed as a potential sources for biofuel production, especially due to their high lipid content [14]. We were interested in determining the response of *Thalassiosira pseudonana* to the various environmental perturbations on its lipid composition. The GC-TOF MS analysis allows measuring only a few low molecular weight lipids, mostly fatty acids and sterols. We have found that the levels of polyunsaturated fatty acid docosahexaenoic acid (DHA), have been elevated in cells deprived of nitrogen and iron (Figure 1). This analyte has been found in the polar phase of the extracts. One might assume that the major amount of this fatty acid can be found in the non-polar phase and that DHA in both phases is in equilibrium. Hence this result, although interesting, should be treated carefully rather as a suggestion of accumulation of this polyunsaturated fatty acid in nutrient-stressed diatoms.

**Figure 4:**
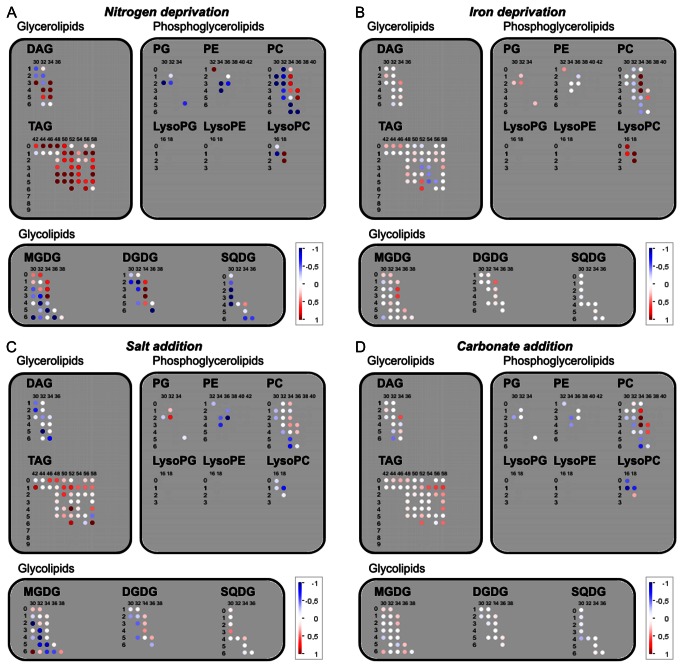
Changes in lipid compounds profiles of*T. pseudonana* grown in different conditions. **A**) Nitrogen deprivation, **B**) lowered iron concentration, **C**) Salt addition, **D**) Carbonate addition. The log_2_-transformed ratios (without statistical significance indication) are visualized as colour spots (red colour marks increase, while blue decrease of an analyte). Two numbers used to describe lipid molecular species: the horizontal number represent total number of carbon atoms in acyl chains, while the vertical number gives the number of unsaturated bonds. Abbreviations: DAG, diacylglycerol; TAG, triacylglycerol; PG, phosphatidylglycerol; PE, phosphatidylethanolamine; PC, phosphatidylcholine; MGDG, monogalactosyldiacylglycerol; DGDG, digalactosyldiacylglycerol; SQDG, sulfoquinovosyldiacylglycerol.

The bulk analysis of lipid compounds could not be performed in the GC-TOF MS and therefore, it was preformed using a recently established liquid chromatography coupled to a high resolution MS-based lipidomics platform [33,34], which allowed us to monitor nine major lipid classes, covering glycerolipids (di- and triacylglycerols), phosphoglycerolipids (phosphatidylcholine, phosphatidylethanolamine, phosphatidylglycerol, lyso-phosphatidylcholine) as well as glycolipids (mono- and digalactosyldiacylglycerols, sulfoquinovosyldiacylglycerols). All together we have measured relative levels of 124 lipid species in *Thalassiosira pseudonana*. An overview of the lipidomic data is displayed in Figure 4. Lipid species are characterized by the class name abbreviation, the number of carbon atoms in their acyl chains (columns in Figure 4) as well as the number of double bonds in the acyl chains (rows in Figure 4).

As expected, lipids with high abundance of 16:1 fatty acids were most abundant in our samples, which is in agreement with earlier reports [13,35,36]. The high abundance observed for diacyl-lipids with 30 C (16 and 14 C fatty acids) and 1 double bound in our results is also finds support in previous reports on fatty acid composition of diatom’s lipids, which indicated that these organisms accumulate more than 15% of their total fatty acids in form of myristic acid [13,35,36].

With respect to the different treatments applied, again nitrogen deprivation had the biggest and most severe influence. The most significant changes concern triacylglycerols (TAG). Triacylglycerols with saturated fatty acids chains (TAG 42: 0 to TAG 50: 0) were most affected, increasing between 1.6- to 5.9-fold as compared to the control nitrogen-replete level (Figure 4). TAG’s with longer and saturated acyl chains (TAG 52: 0 to TAG 58: 0) showed little change, while levels of those containing unsaturated fatty acids were elevated as well (Figure 4). The increase in TAG’s in response to nitrogen limitation has also been observed in case of higher plants and chlorophytes [14] [37] [38].

Other significant changes concern the increase in diacylglycerols (DAG) species, which are known to be either pathway intermediates or signalling molecules. In our case DAGs with higher desaturation degree, were elevated, while a significant decrease in most of the nitrogen-containing phosphatidylethanolamine (PE) and phosphatidylcholine (PC) species was observed (Figure 4).

Interestingly, salt stress also led to the accumulation of TAG species, especially those with low degree of desaturation (Figure 4 D). Further, we have observed decreases in diacyglycerols, phosphatydylcholine, phosphatidylethanolamine and in monogalactosyldiacylglycerols (MGDG). Carbonate addition and lower iron conditions had relatively mild or had no effects on the lipid composition of *Thalassiosira pseudonana*. Most significant changes concern some PC and some LysoPC species.

## Conclusions

Here we present metabolic profiles (primary metabolites and lipids) observed for *Thalassiosira pseudonana* in response to changing environment. The metabolic analysis was complemented by the expression analysis of selected central metabolism genes. The iron deprivation and the carbonate addition had mild effects on the metabolic phenotype of the diatom cultures. On the other hand, the nitrogen deprivation for only 24 hours is a strong stress to the diatom cells leading to accumulation of storage lipids and massive reduction of N-containing metabolites. The responses described should here be of value for understanding of the diatoms adaptation processes to various ecological growth condition or treatments, which is relevant for establishing efficient cultivation procedures for biotechnology.

## Methods

### Growth conditions of the diatoms

*Thalassiosira pseudonana* (accession CCMP 1335) starter culture was obtained from National Center for Marine Algae and Microbiota. Diatoms were maintained in the sterile f/2 medium [39]. To control the chemical composition of the growth conditions, the medium was prepared by dissolving the f/2-salts and vitamins in an artificial seawater, which has been prepared from inorganic salts according to recipe for ESAW by Berges et al. [40]. The light intensity was 80 µmol/m^2^/s and the temperature was kept at 22°C throughout the 16 h day / 8 h night regime. The decision for a fairly high temperature (22 °C) during growth, which might already present a stress for *T. pseudonana*, is largely motivated by the experimental setup, i.e. significantly faster growth rates at 22 °C as compared to 14 °C the more commonly used temperature. For the experiment an inoculum from a stationary culture was transferred into a fresh f/2 medium. After five days in the middle of logarithmic growth, with 800 – 1500*10^3^ cells/ml density, 40 ml of the diatom culture were gently filtered through Durapore® membrane filters Ø40mm, HV 0.45 µm (Millipore). A filter with diatoms was immediately transferred to 40 ml of a test medium. Filters in flasks were gently swirled to release diatoms back into the medium. For specific action of nutrients limitation, diatom cultures were grown in test f/2 media prepared as described above but without iron or nitrogen supplementation. To investigate effects of increased salt levels, the sea salt (Sigma-Aldrich) in amount of 1.75 g/100 ml, which corresponds to approximately 50% of salt concentration in marine water, was added to the cultures. Finally, the increase of carbonate level was obtained by addition of NaHCO_2_ (0.017 g/100 ml) in form of freshly prepared aqueous solution. Controls were transferred to fresh f/2 medium. After 24 hours in the test conditions, diatoms for the dimethylsulfoniopropionate measurements and the transcripts analysis were harvested by filtration. The filters for dimethylsulfoniopropionate (DMSP) analysis were immediately used as described below. The filters with diatoms for the RNA extraction were snap-frozen in liquid nitrogen and stored in -80°C prior to extraction. For the metabolic profiling, the rest of a culture was spun in a 50 ml falcon vial for 5 min at 800 g in 4°C. The pellets were frozen in the liquid nitrogen for storage. Each treatment was conducted in 5 replicates.

### Metabolites measurements

To determine the dimethylsulfoniopropionate (DMSP) content in diatoms an indirect method described by Niki *et al*. [41] was applied. Hydrolysis of DMSP was performed in glass vials closed with screw-caps with silicon septa (Gerstel). Each vial contained 3.75 ml of 1 M NaOH. A 3 ml sample of diatom culture was harvested by filtration (Durapore® membrane filters Ø25mm, GV 0.22 µm; Millipore) and the filter was immediately placed in the neck of a vial containing 1M NaOH. The vials were tightly closed to prevent analyte loss. Finally, the filter was washed into the NaOH by vigorous shaking. Samples were left over night in the dark. Dimethylsulfide released through alkaline lysis was measured using a gas chromatograph coupled to a mass spectrometer (5975B, Agilent Technologies) equipped with MultiPurpose Sampler (Gerstel) for solid-phase microextraction from the head-space of sample vials (HS-SPME). The SPME fibre was coated with a carboxen and polydimethylsiloxane (coating thickness 75 µm). Sampling of DMS was done by exposing SPME fibre in the head-space of the vial for 10 min with agitation at 50°C, followed by thermal desorption (60 s, 250°C) in the injection port of the gas chromatograph. The analyte were separated on GC column J & W DB 624, 250 µm x 60 m (Agilent Technologies) in a stream of helium carrier gas (flow 1 ml/min for 20 min). DMS elution (retention time 8 min) was monitored by 62 m/z and 47 m/z ion mass traces. The integration was made with the MDS ChemStation software provided by Agilent Technologies.

Metabolite extraction form *Thalassiosira pseudonana* was performed by use of method described by Giavalisco et al. [34] and Hummel et al. [33]. In short, cell pellets were extracted with cold mixture of methyl-tert-butyl-ether: methanol (3:1). To facilitate cell disruption samples were incubated in a cooled sonic bath for 10 min. The subsequent addition of a water: methanol (3:1) mixture to the extract resulted in a formation of two liquid phases (polar and non-polar phase). Each phase was aspired, dried in vacuum and kept in -20°C prior to the metabolite profiling.

The polar-sample preparation and derivatization of metabolites for analysis by the GC-MS were performed as outlined by Lisec et al. [42]. The GC-MS data were obtained using an Agilent 7683 series autosampler (Agilent Technologies), coupled to an Agilent 6890 gas chromatograph – Leco Pegasus time-of-flight mass spectrometer. Chromatograms were exported from Leco ChromaTof software (version 3.25) to R software. Peak detection, retention time (RT) alignment and library matching were obtained using the TargetSearch R package from bioconductor [43]. For normalization of data the intensity of each analyte peak was divided by intensity of internal standard (sorbitol) and optic density (OD_600_) as a measure for cell density. In our previous experiments, the optic density of an axenic *T. pseudonana* culture in the mid-log growth phase showed a linear correlation with the cell count measured with CyFlow Space (Partec), a fluorescent assisted cell sorter (Figure S1). Visualization of results was performed in R (boxplots) [44] and heatmaps were generated by MultiExperiment Viewer [45], while lipid data were visualized with use of MapMan [37].

The non-polar phase of *Thalassiosira pseudonana* extracts were analyzed as described by Giavalisco et al. [34]. Fractionation of lipids extract was performed by a Waters Acquity UPLC system using a C_8_ reversed-phase column (100 mm × 2.1 mm × 1.7 μm particles; Waters). The mass spectra were acquired using an Exactive mass spectrometer (Thermo-Fisher). The spectra were recorded alternating between full-scan and all-ion fragmentation-scan modes, covering a mass range from 100 to 1500 m/*z*. Peaks were annotated on basis of their m/z and retention time by comparison with an in-house database [34]. Data were normalized to optic density (OD_600_) of a sampled culture and to median of all measurements values for each analyte.

### Quantitative Real-Time PCR

The extraction of total RNA was performed with use of Trizol reagent (Invitrogene). The filters with frozen *T. pseudonana* were extracted in 700 µl of the reagent and after addition of 200 µl of chloroform, the polar phase was transferred into a new tube. The nucleic acids containing polar phase was mixed with isopropanol (0.6 volumes) and 3M sodium acetate (0.2 volumes) to precipitate RNA in room temperature. The RNA precipitate was washed three times with 70% ethanol. The aqueous solution of RNA was treated with DNase (Frementas) according to manufacturer’s protocol. The cDNA was synthesized from DNA-free RNA by use of oligo-dT_18_ and the RevertAid Premium Reverse Transcriptase (both Fermentas) according to manufacturer’s protocol.

Primers for the PCR were designed in Primer Express 2.0 (Applied Biosystems) basing on the sequences obtained from queries for diatom homologues in the genome database of *Thalassiosira pseudonana* (http://genome.jgi-psf.org/Thaps3/Thaps3.home.html). Settings for primer pair selection were: Primer length: 19-21 bp; T_m_: 59-61°C; Amplicon length 85-110 bp. As a reference an actin (ACT4, 269504) gene was used [9]. Sequences of primers used in this research are in Table S1.

The quantitative Real Time-PCR was conducted with an ABI PRISM^®^ 7900 HT Sequence Detection System (Applied Biosystems) and SYBR^®^ Green (Applied Biosystems) was used to monitor product formation. The PCR conditions were as described by Czechowski *et al*. [46]. The reaction mixture contained 5 μl 2-times SYBR^®^ Green Master Mix reagent (Applied Biosystems), 1 μl of template diatomal cDNA and 1 µl of both forward and reverse gene-specific primers, 1 pmol each. The data were recorded and analysed by SDS 2.3 software (Applied Biosystems).

The linear range of polymerase chain reaction as well as optimal threshold was identified with use of LinReg 11.6 software [47]. Threshold crossing time (C_T_ values) for all genes were normalized to the C_T_ of housekeeping reference gene [9] by subtracting the C_T_-value of the gene of interest from the C_T_-value of actin gene. A relative expression in test conditions was calculated by dividing the measured expression by the median of a gene expression in the control conditions. Data were visualized in R [44].

## Supporting Information

Figure S1 Correlation of the optic density (600 nm) and the chlorophyll-containing particles concentration in *Thalassiosira pseudonana* cultures. A fitted trendline, a correlation coefficient value and an equation used for the cell density estimation are visualised on the plot.

Table S1 Expression levels and primer sequences of analysed genes. The table presents normalized to actin and relative to the control expression levels of selected *T. pseudonana* genes. Statistical significance was assessed by HSD Tukey test and on which basis following symbols were assigned: for P-value > 0.05 "ns"; <0.05 "*"; <0.01 "**".
