# Supplementary figures and images for: Metabolic Analysis of Adaptation to Short-Term Changes in Culture Conditions of the Marine Diatom Thalassiosira pseudonana
Source: PLoS One. 2013 Jun 14;8(6):e67340. doi: 10.1371/journal.pone.0067340 (PMC3682967; doi:10.1371/journal.pone.0067340)

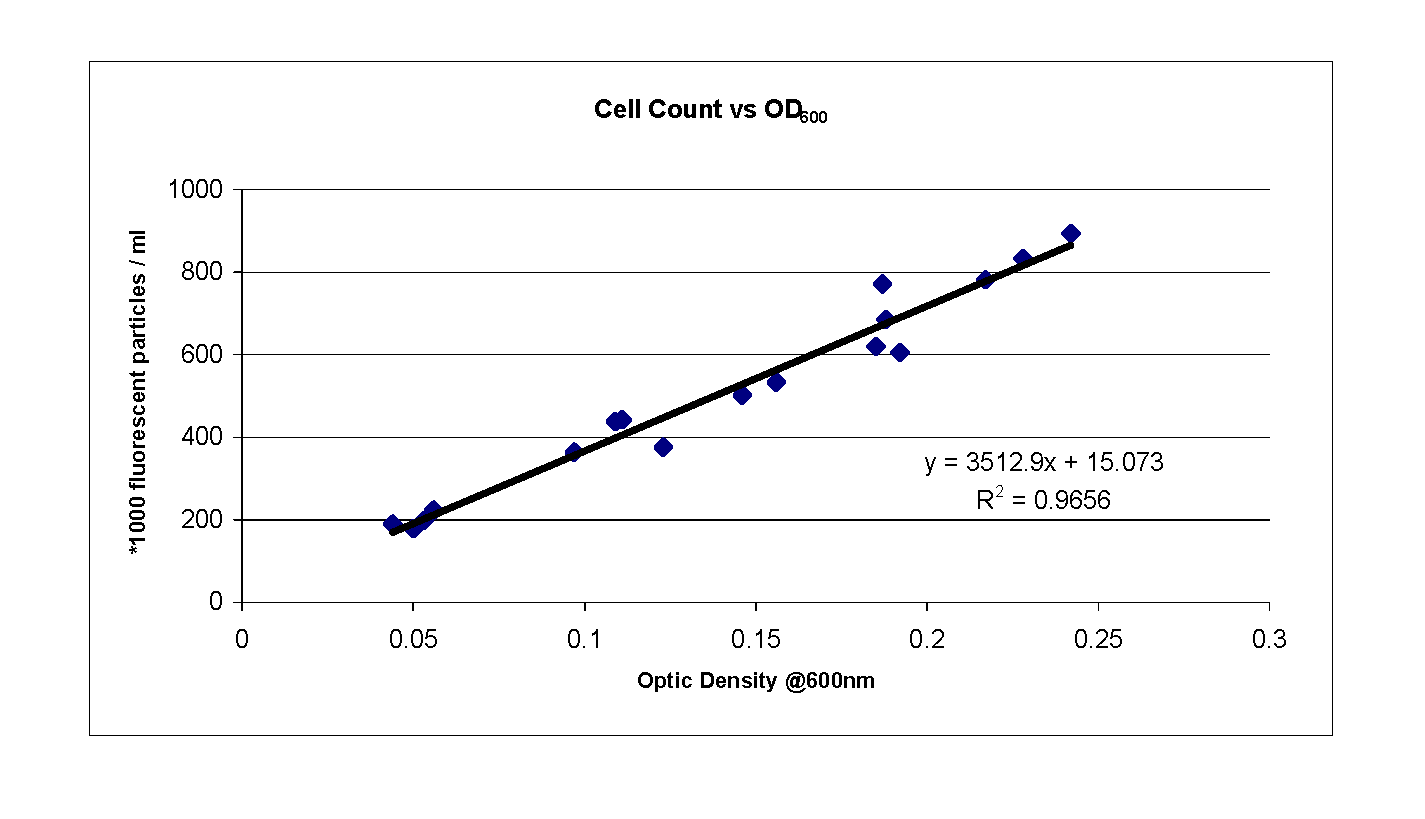

Supplement: Figure S1 — A fitted trendline, a correlation coefficient value and an equation used for the cell density estimation are visualised on the plot. [file pone.0067340.s001.tif]
